# A pedigree‐based experiment reveals variation in salinity and thermal tolerance in the salmon louse, *Lepeophtheirus salmonis*

**DOI:** 10.1111/eva.12505

**Published:** 2017-08-16

**Authors:** Lina Eva Robin Ljungfeldt, María Quintela, François Besnier, Frank Nilsen, Kevin Alan Glover

**Affiliations:** ^1^ Institute of Marine Research Bergen Norway; ^2^ Sea Lice Research Centre Department of Biology University of Bergen Bergen Norway

**Keywords:** aquaculture, evolutionary capacity, marine, microsatellite, parasite, resistance, sea lice, temperature

## Abstract

The salmon louse is a highly abundant ectoparasitic copepod of salmonids in the North Pacific and Atlantic. Widespread and rapid development of resistance to chemical agents used to delouse salmonids on marine farms is now threatening the continued development of the aquaculture industry and have served as a potent catalyst for the development of alternative pest management strategies. These include freshwater and warm‐water treatments to which the louse is sensitive. However, given the well‐documented evolutionary capacity of this species, the risk of developing tolerance towards these environmental treatments cannot be dismissed. Two common‐garden experiments were performed using full‐sibling families of lice identified by DNA parentage testing to investigate whether one of the fundamental premises for evolution, in this context genetic variation in the capacity of coping with fresh or warm water, exists within this species. Significant differences in survival were observed among families in both experiments, although for the salinity experiment, it was not possible to unequivocally disentangle background mortality from treatment‐induced mortality. Thus, our data demonstrate genetic variation in tolerance of warm water and are suggestive of genetic variation in salinity tolerance. We conclude that extensive use of these environmental‐based treatments to delouse salmonids on commercial farms may drive lice towards increased tolerance.

## 1 INTRODUCTION

Human ecological impact has massive evolutionary consequences and can greatly accelerate evolutionary change in many species, namely aqua‐ and agricultural pests, disease organisms or species hunted commercially. Rates of human‐mediated evolutionary change can exceed the natural rates by orders of magnitude (Reznick, Bryga, & Endler, 1990). Furthermore, in species living in human‐dominated systems, rapid evolution in the direction of the human‐induced selection pressure is expected (Hoy, 1998). This conveys the exposure of societies to uncontrollable disease or pest outbreaks, rapid changes in invasive species, life‐history change in commercial fisheries, pest adaptation to biological engineering products, antibiotic and human immunodeficiency virus (HIV) resistance to drugs, or plant and insect resistance to pesticides (e.g. Levy, 1994; Palumbi, 2001; Pimentel & Lehman, 1994; Thompson, Hiatt, Facciotti, Stalker, & Comai, 1987).

In most cases, the evolutionary pattern consists of the following steps: (i) the species is variable for a trait that (ii) confers a difference in survival or production of offspring, and (iii) the trait has an underlying genetic basis. When these requirements are met, the evolutionary engine can turn, even though evolutionary directions and speed can be modified by drift or conflicting selection pressures (Endler, 1986). At this juncture, and considering that our impact on the biosphere is not likely to decline, the evolution in the wake of human ecological change becomes the default prediction and should be incorporate to every analysis when releasing new biocides, health policies or biotechnology products. In addition, planning mechanisms that can help reduce the rate evolutionary change and controlling arms races in disease and pest management can largely reduce our evolutionary impact and ameliorate the economic and social costs of evolution (Ewald, 1994; Lamichhane, Dachbrodt‐Saaydeh, Kudsk, & Messéan, 2015).

The Atlantic salmon (*Salmo salar* L.) aquaculture industry plays a major role in the so‐called global blue revolution (i.e. the emergence of aquaculture as a highly productive way of food supply) and was by far the most valuable cultured fish species in the world in 2014 (14.6 billion USD (FAO 2016)). The rapid development of the salmon aquaculture industry has not been without major challenges, however. Of these, farmed escapees and infestations with the parasitic salmon louse *Lepeophtheirus salmonis* (Krøyer, 1837) are currently regarded as the two most significant issues to environmental sustainability (Glover et al., 2017; Taranger et al., 2015).

The salmon louse is a ubiquitous marine ectoparasite of salmonids in the Northern Hemisphere (Kabata, 1979, 2003) and is divided into the Pacific *L. salmonis oncorhynchi* and the Atlantic *L. salmonis salmonis* subspecies (Skern‐Mauritzen, Torrissen, & Glover, 2014). Salmon lice display a high reproductive output, releasing large numbers of planktonic larvae into the surrounding water masses that are thereafter spread via the marine currents. These infect farmed salmonids reared in cages (Torrissen et al., 2013), wild Atlantic salmon postsmolts migrating to offshore areas, as well as wild sea trout (*Salmo trutta*) and Arctic charr (*Salvelinus alpinus*) that stay in coastal waters (Finstad & Bjørn, 2011; Heuch & Mo, 2001; Heuch et al., 2005; Jones & Beamish, 2011). High levels of infection in both farmed and wild hosts can inflict extensive physiological problems, and ultimately death (Wagner, Fast, & Johnson, 2008). Control procedures on commercial farms have relied extensively upon the use of chemotherapeutants for more than two decades (Boxaspen, 2006; Brooks, 2009; Pike & Wadsworth, 1999). However, lice have evolved resistance to most of these agents (Denholm et al., 2002; Fallang, Denholm, Horsberg, & Williamson, 2005; Fallang et al., 2004; Sevatdal, Copley, Wallace, Jackson, & Horsberg, 2005), in particular to organophosphates, pyrethroids and emamectin benzoate (Besnier et al., 2014; Espedal, Glover, Horsberg, & Nilsen, 2013; Jones, Hammell, Gettinby, & Revie, 2013; Jones, Sommerville, & Wootten, 1992; Ljungfeldt, Espedal, Nilsen, Skern‐Mauritzen, & Glover, 2014; Sevatdal & Horsberg, 2003). The loss of efficiency of these treatments has served as a potent catalyst to develop and implement alternative delousing procedures in aquaculture (Lekang, Salas‐Bringas, & Bostock, 2016), including warm‐water (Havardsson, 2013) and freshwater treatments (Grøntvedt et al., 2015; Reynolds, 2013) to which lice are sensitive at the present.

Salinity is known to have direct and indirect effects on survival, metabolism, growth, reproduction and osmotic balance in aquatic crustaceans (e.g. Chand et al., 2015; Jian‐Wen & Pei‐Yuan, 1999; Łapucki & Normant, 2008; Normant & Gibowicz, 2008; Normant & Lamprecht, 2006; Whiteley, Scott, Breeze, & McCann, 2001). The salmon louse shows optimal survival and development at salinities greater than 27 ‰ (Bricknell, Dalesman, O'Shea, Pert, & Luntz, 2006). However, some adult females not attached to a host can osmoregulate down to 12.5 ‰ (<8 hr to death in freshwater), and some individuals have been reported to survive in freshwater up to 14 days when attached to a host, probably assisted through the acquisition of diet‐obtained ions (Connors, Juarez‐Colunga, & Dill, 2008; Hahnenkamp & Fyhn, 1985). Nevertheless, despite the capacity for some adults to survive several days in lower salinities and freshwater (see also Pike & Wadsworth, 1999), it has also been reported that parasite infestation is lower on fish collected from zones with lowest sea water surface salinity (Jones & Hargreaves, 2007). Whether or not genetics plays a role in this variation remains, however, unknown.

Temperature influences all physiological processes from the molecular level to that of the whole organism (Angilletta, 2009; Kingsolver, Ragland, & Diamond, 2009). In addition, it exerts a profound impact on the structure, dynamics and functioning of populations (Angilletta, 2009; Dillon, Wang, & Huey, 2010; Morelissen & Harley, 2007). Thus, as for most ectotherms, water temperature displays a causative relationship with developmental time, adult body size and reproductive output in the salmon louse (Angilletta, Steury, & Sears, 2004). The body of literature investigating the effect of temperature on different life‐history traits in the salmon louse includes topics such as time to hatch (Boxaspen, 2006; Boxaspen & Naess, 2000; Costello, 2006; Johnson, Treasurer, Bravo, Nagasawa, & Kabata, 2004), egg viability (Johnson & Albright, 1991), settlement and survival of copepodids (Tucker, Sommerville, & Wootten, 2000a,b), developmental rate (Samsing et al., 2016; Tucker et al., 2000a,b), larval development (Boxaspen & Naess, 2000; Brooks, 2005; Pike & Wadsworth, 1999), body size (Samsing et al., 2016), maturation (Stien, Bjørn, Heuch, & Elston, 2005), mortality (Bricknell et al., 2006; Johnson & Albright, 1991) and infestation rate (Costello, 2006; Jones & Hargreaves, 2007).

An essential step for the effective management of the salmon louse within commercial aquaculture is to understand the influence of changes in environmental conditions on the propagation dynamics of louse populations (Brooks, 2005, 2009; Price, Morton, & Reynolds, 2010). Given the high reproductive output, short generation time and very high abundance of this species, the potential for rapid evolution, including human‐induced selection regimes, is foreseeable. In this context, the emerging use of unfavourable environmental conditions as a nonchemical alternative strategy to treat lice infestations on farmed salmonids (i.e. treating infested fish with low salinity or high‐temperature water [e.g. Havardsson, 2013; Reynolds, 2013; Grøntvedt et al., 2015]) could provide a strong source of selection if genetic variation for tolerance to either of these environmental‐based treatments exists.

Ljungfeldt et al. (2014) published the first pedigree‐based “common‐garden” experiment with a copepod using an approach that included synchronized production of full‐sibling salmon lice families, exposure to a challenge (a chemotherapeutant), sorting by phenotypic response (dead/alive), and DNA parentage testing to compare family performance as a proxy for genetic variation. That experimental set‐up managed to prove the existence of genetic variation in the resistance to the delousing chemical emamectin benzoate, a result that was subsequently validated at the genomic level using a SNP chip and linkage mapping on the samples originating from the initial common‐garden experiment (Besnier et al., 2014). In the present study, we used the protocol and infrastructure established by Ljungfeldt et al. (2014) to quantify family differences (as a proxy for genetic variation) in tolerance to a low salinity and a heat challenge. Ultimately, this was to evaluate whether the emerging practice within the commercial aquaculture industry of delousing farmed salmonids with fresh‐ and/or warm‐water treatments may elicit an evolutionary response in this parasite and lead to reduced treatment effectiveness.

## 2 MATERIAL AND METHODS

### 2.1 Overall experimental design for both experiments

**Figure 1:**
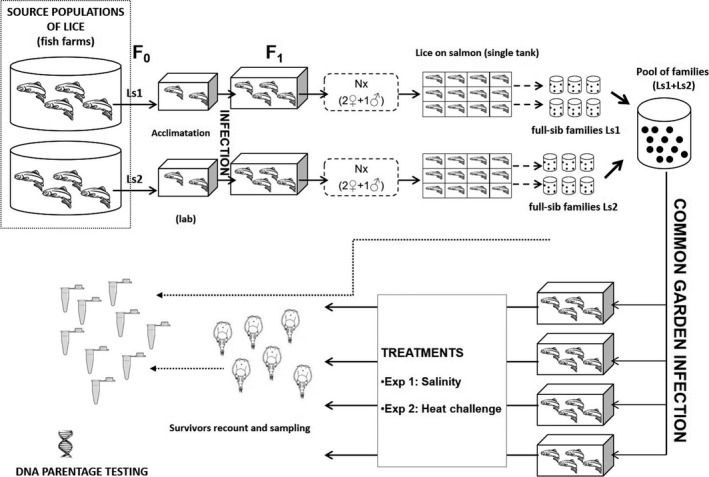
Outline of the overall experimental procedure used for experiments 1 (salinity challenge) and 2 (heat challenge). Ls1, 2 =  lice strain 1, 2, respectively

Two separate experiments, a low salinity and a heat challenge, were conducted. Both experiments follow the overall experimental design detailed in Ljungfeldt et al. (2014), which includes the following steps (Figure 1) (i) Acquisition of two strains of salmon lice, *L. salmonis salmonis*, from fish farms situated in two different salinity/thermal environments, respectively. (ii) Synchronized production of single‐strain parental populations. (iii) Synchronized creation of full‐sibling families to be mixed in a common pool. (iv) Common‐garden infection in replicate salmon tanks with an exact number of copepodids from each of the families. (v) Experimental treatment of lice (salinity or heat challenge). (vi) Sampling of lice sorted by trial response (survivors *vs*. nonsurvivors). (vii) Individual genotyping of parents and sampled offspring for family identification and subsequent quantification of family performance (as a proxy for potential genetic variation). It is important to note that we established lice strains originating from two contrasting salinity/thermal environments in each experiment, respectively. This was done in order to increase the potential for observing genetic variation for the target traits, and not to test the potential for habitat‐driven adaptation.

### 2.2 Animal welfare considerations and rearing conditions

Salmon lice belong to the systematic entities that are not protected by animal welfare legislation, but their development past the infective copepodid stage requires attachment to a salmonid host. Thus, the Norwegian Animal Welfare Act regulations for the maintenance of host fish have been followed to conduct these studies under permit number 2009/186329.

### 2.3 Experiment 1: Salinity

#### 2.3.1 Genetic background of the lice used in the salinity experiment

Two strains of salmon lice were obtained from fish farms located in different salinity regimes. The full salinity strain (LsS) was founded upon lice collected from Atlantic salmon sampled on a farm in Øygarden municipality (60°34′ 24″N; 4°49′ 0″E) in Hordaland county, western Norway. This represents a euhaline (ppt>30) coastal site, exposed to constant high salinity. The brackish strain (LsB) was founded upon lice collected at a rainbow trout (*Oncorhynchus mykiss*) farm in Osterfjorden (60°31′ 33″N; 5°21′ 26″E), also in Hordaland. This farm is located in a polyhaline (ppt 13‐30) fjord with lower and more variable salinity levels, due to freshwater run‐off from precipitation and snow melting.

#### 2.3.2 Production of lice families for the salinityexperiment

Pairs of egg strings from 94 LsS females and 113 LsB females were collected, incubated, and after 14 days, used for two single‐strain (F1 generation) infections in two separate tanks containing 25 salmon each. The F1 generation lice were collected on 36 DPI (days postinfection) and placed on fish (one adult male and two pre‐adult II females, i.e. virgins, per fish) in single fish tanks to establish full‐sibling families from each strain with complete control over parentage and no opportunity for multiple parentage. Once the fertilized females from the F1 generation had produced their second sets of egg strings, these were collected (F2 generation) and incubated in single‐family incubators. At the time of selecting families for the common‐garden infection (one family per male to avoid half‐sibs in the paternal line), the copepodid clutches were of variable ages, 6–15 days posthatching (DPH), due to the naturally occurring variation in egg sac development among females (Gravil, 1996a,b). Lower infection success for ageing copepodids (Gravil, 1996a,b; Tucker, 1998) has been linked to impaired attachment capability as a result of gradual depletion of the energy reserves (Tucker et al., 2000a,b). Under our experimental conditions, this stage of senescence corresponds to 10 DPH; therefore, only families in the range 6–9 DPH were retained for the common‐garden infection.

#### 2.3.3 Common‐garden design for the salinityexperiment

The common‐garden experiment was conducted in four replicate tanks, each containing fifteen salmon. A total of 4,943 copepodids, ranging from 205–596 from each of twelve full‐sibling families (representing the F2 generation from both founder strains), were used to infect the four replicate tanks. To control for background mortality of the lice pre‐ and during the low‐salinity challenge, filters were placed on the outlets of all four tanks and inspected for detached lice daily. On day 63 PI, the salinity was gradually decreased over 1 hr from 34.5‰ to ≈13‰ in all four replicate tanks. The following day, salinity was increased slightly to ≈15‰ and held constant for twelve days until the experiment was terminated (~640 degree‐days postinfection at a mean (±*SD*) temperature of 8.45 ± 0.42°C). Upon termination, fish were anesthetized for 2–3 min in a mixture of metomidate (5 mg/L) and benzocaine (60 mg/L) in sea water, one fish at a time. Lice were removed and sorted according to sex and the presence of egg strings. All individuals sampled upon termination of the experiment were stored for subsequent DNA parentage testing.

Low salinity is known to reduce hatching success and hamper development due larval limited capacity for osmoregulation (Bricknell et al., 2006; Bron, Sommerville, Wootten, & Rae, 1993; Gravil, 1996a,b). To assess whether there was any difference among families regarding egg viability, we collected one hundred egg strings that were incubated individually in full sea water for a maximum of 20 days, and monitored daily. Hatchlings were examined 8–9 DPH, when they should have developed into copepodids under the current rearing conditions.

### 2.4 Experiment 2: Heat challenge

#### 2.4.1 Genetic background of the lice used in the heat challenge experiment

Two strains of lice were obtained from Atlantic salmon farms located in different temperature environments some 1,500 km apart (flying distance, see Fig. S1). The northern strain (LsNo) was founded upon lice collected on a farm located in Kvalsund municipality (70°23′ 10″N; 23°28′ 20″E) in Finnmark, the northernmost county in Norway with an annual average sea water temperature (±*SD*) of 6.93 ± 2.19°C. The southern strain (LsSo) was founded upon lice from a salmon farm in Hyllestad (Sogn og Fjordane) (61°12′ 6″N; 5°10′ 15″E) with an annual average sea water temperature (±*SD*) of 9.69 ± 3.55°C (Fig. [Link], [Link]).

#### 2.4.2 Production of lice families for the heat challenge experiment

Pairs of egg strings from 183 LsNo females and 189 LsSo females were collected, incubated and, 14 days later, used for single‐strain (F1 generation) infections on 18 salmon in two separate tanks. From this initial infection, thirty‐five DPI (days postinfection) lice were collected and put on fish (one adult male and two pre‐adult females) in 33 single fish tanks (i.e. one fish per tank as in the salinity experiment). From a total of 66 families established, 15 were selected for the common‐garden experiment as follows: five pure strain LsSo, five pure strain LsNo and five hybrid LsNo x LsSo families (*N* = 3 produced by pairing LsNo females and LsSo males and *N* = 2 LsNo males with LsSo females).

#### 2.4.3 Common‐garden design for the heat challenge experiment

Before the common‐garden experiment was conducted, a pilot study was carried out in order to establish a protocol which enabled exposing the lice in vitro to a heat challenge that would cause selective mortality in a predictable and accurate manner, but simultaneously enabled dead lice to be rapidly sampled into EtOH to ensure DNA quality enabling parentage testing. The pilot test, including its results, is described in full in the Supplementary File. This gave rise to the below protocol.

The heat challenge experiment was conducted by mixing all copepodids (*N* = 6,601) from 15 experimental families (ranging from 246–579 per family, Table 1) and thereafter infecting 68 Atlantic salmon equally distributed between four replicate tanks (17 salmon/tank). After the infection, lice were left to develop on the fish at 8.9 ± 0.5°C (mean ±*SD*) for 36 days. This timing was to ensure that, at the time of the heat challenge, the majority of the lice would be of the same size, taking into account the staggered developmental time and size differences between males and females (i.e. most of the females at the second pre‐adult stage and males at the adult stage, see Fig. S3 in Supplementary Material for detailed explanation). The aim was to avoid the potential confounding factors of size or age when assessing survival in relation to sex.

**Table 1:** Survival rates for 15 full‐sibling families in response to the heat challenge experiment. N cops (number of individuals that went to the common pool), N lice sampled from the fish, no lice trial (number of individuals used in the heat challenge experiment) and results for survival after all trials per tank (AAH, i.e. “attached after heat challenges”). Number and (percentage) of survivors are given on a family basis per tank

Family‐ID	Family origin	*N* cops	*N* lice sampled^a^	*N* lice trial	AAH			
					Tank 1	Tank 2	Tank 3	Tank 4
Family‐1	LsNo ♀ x LsNo ♂	246	11	11	0 (0.0)	4 (36.4)	1 (9.1)	1 (9.1)
Family‐2		324	75	73	13 (17.8)	10 (13.7)	15 (20.5)	12 (16.4)
Family‐3		397	167	167	25 (15.0)	31 (18.6)	32 (19.2)	38 (22.8)
Family‐4		393	69	69	13 (18.8)	10 (14.5)	14 (20.3)	14 (20.3)
Family‐5		579	90	87	7 (8.0)	13 (14.9)	16 (18.4)	21 (24.1)
Family‐6	LsSo ♀ x LsNo ♂	272	79	79	8 (10.1)	10 (12.7)	5 (6.3)	16 (20.3)
Family‐7		526	170	166	27 (16.3)	23 (13.9)	38 (22.9)	31 (18.7)
Family‐8	LsNo ♀ x LsSo ♂	476	152	150	20 (13.3)	21 (14.0)	21 (14.0)	21 (14.0)
Family‐9		562	112	112	16 (14.3)	11 (9.8)	22 (19.6)	24 (21.4)
Family‐10		567	199	196	16 (8.2)	41 (20.9)	35 (17.9)	33 (16.8)
Family‐11	LsSo ♀ x LsSo ♂	401	136	135	18 (13.3)	15 (11.1)	22 (16.3)	18 (13.3)
Family‐12		437	85	81	4 (4.9)	7 (8.6)	16 (19.8)	14 (17.3)
Family‐13		465	126	122	12 (9.8)	22 (18.0)	31 (25.4)	28 (23.0)
Family‐14		443	121	120	18 (15.0)	17 (14.2)	24 (20.0)	19 (15.8)
Family‐15		513	134	133	17 (12.8)	31 (23.3)	19 (14.3)	26 (19.5)
Total		6,601	1,726^a^	1,701	214	266	311	316

a The total number of lice sampled from fish was 1,733, but seven of them could not be identified back to family.

In contrast to the salinity experiment, the heat challenge was conducted in vitro, and hence, all lice (*N* = 1,733) were plucked off the salmon hosts and transferred to four oxygenated 3‐L beakers (one beaker per tank). The beakers were held at 9°C in a water bath for 24 hr prior to assessing sampling damage in lice (i.e. individuals injured during the physical removal from fish). After this time, 25 dead lice were discarded from the experiment and registered as “Excl” (excluded from the trial). The remaining lice were exposed to a rapidly increasing temperature as warm water was added to the water bath surrounding the beakers. Water temperatures in the beakers were logged at 30‐second intervals during the entire process, using four TidbIT^®^ v2 temperature loggers from Onset Computer Corporation. The first heat challenge consisted of a rapid increase in temperature 9°C–22°C during 30 min followed by 3.5 hr at ≈22°C. Afterwards, the water in the beakers was gently vortexed and poured out into another container. The water in the trial beaker was replaced with the same volume of ≈10°C sea water. The lice present in the poured‐out water were categorized as “detached at first heat challenge” (DFH), whereas the ones still attached to the beaker walls were categorized as “alive”. After 20 min at ≈10°C, the lice still attached to the beaker (the survivors of the treatment) were exposed to a second heat challenge consisting of a rapid heating up to 24–26°C over 30 min and sorted again into “detached”/”alive” following the former procedure (see Table 2). All lice were immediately transferred to EtOH to preserve the DNA integrity for genotyping. Oxygen concentration in the beakers was logged at ca 30‐minute intervals during the entire process, to ensure that the survival of lice was not hampered by oxygen depletion.

**Table 2:** Summary of results from the heat challenge experiment (data from all 15 full‐sibling families pooled)

Categories	Tank 1	Tank 2	Tank 3	Tank 4
Excl	3 (1.14)	7 (1.64)	5 (0.93)	10 (1.97)
DFH	18 (6.84)	30 (7.03)	129 (24.07)	86 (16.96)
DSH	28 (10.65)	124 (29.04)	91 (16.98)	94 (18.54)
AAH	214 (81.37)	266 (62.30)	311 (58.02)	317 (62.52)
Total	263 (100)	427 (100)	536 (100)	507 (100)

The numbers refer to the total (and percentage) number of lice per tank sampled at the following sampling points: Excl stands for those individuals that were excluded from the trial (i.e. lice wounded or dead during manual removal from the host salmon *N* = 25). DFH depicts “detached at first heat challenge” (i.e. lice detached from the beaker walls after the first heat challenge event). DSH stands for “detached at second heat challenge” (i.e. lice surviving the first heat challenge but detaching from beaker walls at the second one). Finally, AAH means “attached after heat challenges” (i.e. survivors). The number of trial lice was *N* = 1,708 (DFH + DSH + AAH) from an initial number of lice removed from salmon of 1,733.

### 2.5 Genotying and parent testing

All offspring sampled in the salinity and heat challenge experiments were identified back to their family of origin by screening highly polymorphic microsatellite loci and matching their multilocus genotypic profiles to pairs of parents using the genotype exclusion‐based family assignment program FAP v. 3.6 (Taggart, 2007; ). DNA was extracted in a 96‐well format using Qiagen DNeasy kit. Individuals were genotyped at sixteen loci multiplexed in three reactions: multiplex 1 = LsalSTA1, LsalSTA2, LsalSTA4, LsalSTA5 (Todd, Walker, Ritchie, Graves, & Walker, 2004) and LsNUIG14 adapted by Todd et al. (2004); multiplex 2 = Lsal103EUVC, Lsal109EUVC, Lsal110EUVC, Lsal111EUVC (Messmer et al., 2011) and LsNUIG09 (Nolan et al., 2000); and multiplex 3 = Lsal104EUVC, Lsal105EUVC, Lsal106EUVC, Lsal108EUVC (Messmer et al., 2011), LsalSTA3 (Todd et al., 2004) and LsNUIG35B (Nolan & Powell, 2009). Amplification conditions were identical to those described in Glover et al., 2011 and are available from the authors upon request. PCR fragments were separated on an ABI 3730XL sequencer and sized relative to the GeneScan^™^ 500LIZ^™^ size standard (Applied Biosystems). Alleles were scored twice by independent observers, following automatic binning implemented in the Genemapper (v. 4.0) software.

### 2.6 Data analysis

In the salinity experiment, only the survivors (individuals that were alive after the trial) were available for sampling and sex determination. Thus, we tested the effect of type (LsS *vs*. LsB) on the survival rate of the lice families using generalized linear mixed models (GLMM) implemented in the glmer function from the R package lme4 (Bates, Mächler, Bolker, & Walker, 2015) with a binomial distribution. Replicate (tank) and sex were also considered as random intercepts. Given that the age of the copepodids (DPH) seemed to be influential, we also corrected for fixed DPH effect.

In the heat challenge experiment, each individual dead or alive after trial was sampled. We also used a GLMM with binomial distribution and logit link function to model the state of the individual (AAH or DFH) as a binary response to effects of type (LsNo *vs*. LsSo) and family. Replicate (tank) and sex were also considered as random factors. All data analyses were performed in R (R Core Team, 2015). Interactions between factors were not considered due to data set providing too little power for the estimation of interaction terms.

## 3 RESULTS

### 3.1 Experiment 1: Salinity

A total of 622 lice (146–161 per tank) survived the salinity trial, and where thereafter identified to family using DNA parentage testing (this equates to 18.1% of the initial number of copepodids used to infect the fish). The male/female ratio ranged from 1.1–1.3 per tank, and thus, sex was shown to have a moderate, but significant, influence on survival (χ^2^ = 10.5, *p* = .001). The average salinity during the twelve days of low salinity regime ranged between 15.5 and 16.3 ‰ across the four replicate tanks; however, this small variation did not influence survival (χ^2^ = 1.89, *p* = .59).

The families obtained from the most recently moulted copepodids (Fam‐LsB11, Fam‐LsB13, Fam‐LsB14 and Fam‐LsS12) all showed a very low survival (Table 3), and hence, the age of the copepodids (DPH) was proven to have a significant influence (χ^2^ = 17.2, *p* < .001) as the 6‐DPH families showed lower numbers of alive individuals at the end of the experiment. However, and according to the literature (see Frenzl, 2014; Tucker, 1998), this low survival most likely reflects the limited infective capacity of the newly moulted copepodids, and therefore, the remaining analyses were conducted by correcting for the number of days posthatching in the statistical model (DPH).

**Table 3:** Survival rates for 12 full‐sibling families in response to the salinity experiment (data pooled across 4 replicates)

Family	Strain	DPH	N_0_ (cops)	*n* S	S (%)
Fam‐LsB11	R	6	500	1	0.2
Fam‐LsB13	R	6	370	1	0.3
Fam‐LsB14	R	6	205	1	0.5
Fam‐LsB09	R	7	596	92	15.4
Fam‐LsB10	R	7	454	84	18.5
Fam‐LsB12	R	9	358	35	9.8
Fam‐LsS12	S	6	437	37	8.5
Fam‐LsS09	S	7	511	47	9.2
Fam‐LsS10	S	7	337	62	18.4
Fam‐LsS11	S	7	327	50	15.3
Fam‐LsS13	S	7	391	59	15.1
Fam‐LsS14	S	8	457	193	42.2

DPH stands for “days posthatching” and describes the age of copepodids at infection time, N_0_ (cops) corresponds to the initial number of copepodids at infection; *n* S is the number of survivors at termination; and S is the percentage of survival.

**Figure 2:**
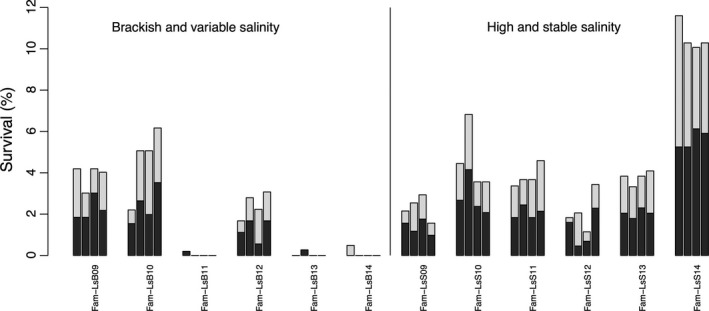
Salinity challenge: percentage of lice surviving the low‐salinity treatment by family. Families belonging to LsB strain originate from variable salinity environment, whereas families belonging to LsS strain originate from high and stable salinity environment. Bars from left to right within family correspond to tanks 1 to 4. Total number of survivors per family can be found in Table 3. The black section of the bars depicts the males, whereas the white one depicts the females. The effect of family on survival revealed a strong and significant variation (*F* = 30, *p* < .001), with Fam‐LsS14 (42% of survivors) showing a substantially higher survival

The percentage survival per family showed a symmetric distribution between LsS and LsB strains of lice in the range 9.2%–18.5% (Table 3, Figure 2). Fam‐LsS14 displayed significantly higher survival than all other families (42%), thus revealing a strong and significant effect of family on survival (χ^2^ = 68, *p* < .001). However, we found no significant differentiation (χ^2^ = 0.0, *p* = .99) between the survival of lice coming from the farm located at a site of high salinity from those originated from the farm located in the inner fjord at lower and more variable salinity conditions. When fitting family tank, sex and strain as random covariates in the same GLMM model, the estimation of the survival variance associated with each covariate was 95%, 5% for family and sex, respectively, while strain and tank had no contribution to the survival variance (Table S2).

The total survival per sex was 55.1% for males *vs*. 44.9% for females. The sex ratio of survivors was not influenced neither by the family the lice belonged to (*F* = 0.42, *p* = .87), nor by strain (*F* = 0.35. *p* = .55), nor by tank (*F* = 0.23, *p* = .87). Likewise, neither strain nor family had a significant influence on egg‐string length (*F* = 0.65, *p* = .42 and *F* = 2.0, *p* = .09, respectively).

To assess the potential impact of freshwater on hatching and larval development, we collected 100 egg strings from surviving females and incubated them. We observed that hatching was unsuccessful in 69 of them and, from a total of 1,688 nauplii observed, only 54 of them (3.20%) were alive and none of them managed to moult into the copepodid stage. The highest numbers of nauplii were produced by Fam‐LsS14 (404) and Fam‐LsS11 (388), the latter one also having the highest survival rate (8%). No eggs from family LsS12 (a six DPH family) managed to hatch (Table S1 in Supplementary material).

### 3.2 Experiment 2: Heat challenge

In the pilot test, only 14 of 609 lice (3.2%) that looked alive immediately after the heat challenge and therefore categorized as survivors, died within the next 24 hr (AD category, Table S2). In contrast, 161 of 609 lice (26.4%) that were categorized as dead following the heat challenge managed to recover in the following 24 hr (DA category, Table S2). As detached lice would not get the opportunity to re‐attach to a host when commercial heat challenge is conducted to delouse fish on farms, this protocol was deemed to be sufficiently accurate to use for the main heat challenge challenge.

**Figure 3:**
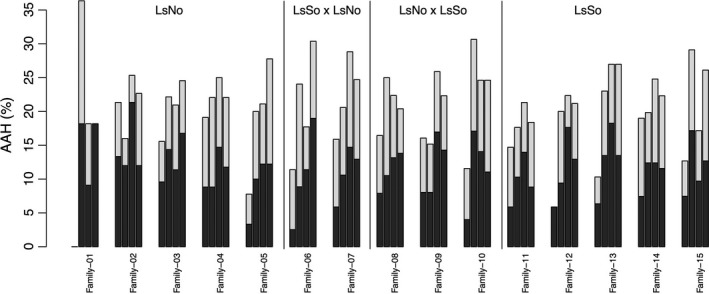
Heat challenge experiment: percentage of lice per family that survived the full heat challenge (i.e. AAH, percentage of lice that stayed “attached after heat challenges”). Bars from left to right within family correspond to tanks 1 to 4. The black section of the bars depicts the males, whereas the white one depicts the females. Families have been sorted according strain origin (pure LsNo, hybrids LsSo x LsNo and LsNo x LsSo, and pure LsSo). Numbers of survivor per tank and family can be found in Table 1

The percentage of lice surviving the full heat challenge (AAH in Table 2) ranged between 58 and 81.4% per tank (Figure 3), and thus, survival rate was significantly different among tanks (χ^2^=61, *p* < .0001). Sex was also associated with survival (χ^2^=82, *p* < .0001), with females performing slightly better than males (survival rate of 88% *vs*. 81%, respectively).

When investigating the effect of strain and families on survival, the strain (LsNo, LsSo or Hybrid) was significantly associated with survival (χ^2^=4.5 *p* = .03) with a small advantage for the hybrids while there was no significant difference of survival rate between LsNo and LsSo. The survival rate was correlated with among‐family variation (χ^2^=16, *p* < .0001) and stronger that the effect of strain. When fitting family tank, sex and strain as random covariates in the same GLMM model, the estimation of the survival variance associated with each covariate was 22%, 56% and 20 for family tank and sex, respectively, while strain had no contribution to the survival variance (Table S2).

## 4 DISCUSSION

To our knowledge, this is the first pedigree‐based study of environmental tolerance in any species of copepod. It was primarily designed to investigate the potential for genetic variation in tolerance to low salinity and a heat challenge in the salmon louse, an economically and ecologically highly significant parasite of farmed and wild salmonids in the North Atlantic and Pacific oceans. In both the salinity and heat challenge experiments, highly significant differences in family survival were observed. While the underlying difference in family survival was not unequivocally disentangled from the treatment effect in the low‐salinity experiment (but see mitigating discussion of this below), background mortality was accurately controlled for in the heat challenge. Therefore, our results demonstrate that genetic variation occurs in salmon lice for heat tolerance, and suggest the same for salinity tolerance. Given that freshwater and high‐temperature treatments are currently being employed within commercial aquaculture to delouse farmed salmonids infected with chemical‐resistant lice (e.g. Grøntvedt et al., 2015; Havardsson, 2013; Reynolds, 2013), our data clearly demonstrate the potential for this parasite to develop reduced sensitivity to these environmental‐based treatments also.

### 4.1 Salinity challenge

The tolerance of marine copepods to differing and changing salinities has been investigated both in laboratory experiments (e.g. Bravo, Pozo, & Silva, 2008; Damgaard & Davenport, 1994; Jian‐Wen & Pei‐Yuan, 1999; Lance, 1963) and in the wild (e.g. Selifonova, 2009, 2011; Svetlichny & Hubareva, 2014). However, the uniqueness of the present study resides in the implementation of a pedigree‐based approach to identify any potential genetic component related to salinity tolerance. The adaptive consequence of genetic variation in salinity tolerance has been documented in marine copepods. For instance, the coastal species *Acartia tonsa* and *Oithona davisae* both managed to establish self‐sustaining populations in low‐salinity estuarine habitats in the Black Sea after transfer in ship ballast water (Gubanova et al., 2014). Likewise, the copepod *Eurytemora affinis* has made the transition from marine to freshwater habitat relatively rapid, demonstrating a large shift in the ability to osmoregulate (Lee, Posavi, & Charmantier, 2012). The rapid adaptation to new environments could suggest the pre‐existence of genetic variation for salinity tolerance in these species.

The lower limit of optimal salinity for adult *L. salmonis* has been reported to be 16 ‰ at a temperature of 14–15°C (Berger, 1970). Still, it has been shown that adult females without a host can osmoregulate down to 12.5 ‰ salinity (<8 hr to death in freshwater), and some can survive in freshwater up to 14 days when attached to a host, possibly through diet‐obtained ions from the host (Connors et al., 2008; Hahnenkamp & Fyhn, 1985). Salmon lice on juvenile Pacific salmon *Oncorhynchus gorbuscha* and *O. keta* were predicted an average survival time of 14.46 ± 2.29 days at 14‰ (Connors et al., 2008). In our experiment, lice were challenged for 13 days at ~15‰ (i.e. below the lower optimal salinity level) and revealed highly variable survival among families, with one of the families (Fam‐LsS14) showing a substantially higher survival (42% vs. 9%–19%, Table 3).

The present study was designed to investigate family differences, as a proxy for genetic variation, in the ability to cope with salinity at a lower level than had been previously reported to cause mortality in this species when attached to a host (Connors et al., 2008). While large differences in family survival were observed in the challenge, thus suggesting genetic variation for salinity tolerance, isolating the effect of salinity on the family performance was hampered by some potentially confounding factors. Lice were exposed to the low salinity challenge while still attached to the hosts (in contrast to the heat challenge). Consequently, it was not possible to unequivocally disentangle background mortality (also called the “invisible fraction”, see Grafen (1988) and Hadfield (2008)) from the mortality specifically induced by the low‐salinity treatment itself. We attempted to address this by placing filters on the outlet of all tanks to retrieve detached lice each day (this would have enabled fractioning background and salinity mortality). However, the filters functioned very poorly, capturing only a few lice (detached lice in tanks are often eaten by their hosts (Connors et al., 2008)). Nevertheless, the overall mortality in the salinity challenge (86.6%) was higher than the background mortality identified by Ljungfeldt et al. (2014) (57.5%) and the background mortality observed in the heat challenge experiment presented here (73.7%). Thus, while it is not possible to completely disentangle background and salinity‐induced mortality, all available evidence suggests that salinity reduced lice survival in this experiment and therefore contributed to the significant differences in survival observed among the families.

As full‐sibling families were used for the present experiment, maternal, dominance and/or epigenetic effects could have influenced family survival in addition to genetic variation for salinity tolerance (and temperature). We minimized such potential effects a) using lice families produced from synchronized strains that had been laboratory‐reared under controlled identical conditions for >1 generation (see Figure 1); and b) by correcting for the effect of the age of copepodids (DPH) on survival. In this context, Frenzl (2014) reported a severe attachment incapability in freshly moulted copepodids (0 days postmoult, DPM, corresponding to 6 DPH in our study), whereas the infection ability remained constant between 1 and 5 days DPM *(*i.e. corresponding to our window of 7–9 DPH). The extremely low survival in the 6 DPH families in our study (0.2%–8.5% survival, Table 3) was thus most likely to be the result of the low attachment ability of the newly moulted copepodids rather than the effect of the low salinity. This infectivity time span is poorly documented in the literature, but according to the results presented here, plays a vital role for infection success. After correcting for the age of the copepodids, we found a significant difference in family survival in the low salinity experiment, primarily driven by the high survival of family LsS14 (across all four replicates). Thus, while potentially confounding effects were present in the salinity challenge, these data indicate genetic variation for tolerance of low salinity. This result is consistent with the results of Bengtsen, Asplin, Bjørn, and Sundby (2012) who observed individual lice tolerating salinities down to 10 ‰, and results from other studies demonstrating the ability of adult lice attached to its host to osmoregulate (Connors et al., 2008; Hahnenkamp & Fyhn, 1985).

The salinity experiment was followed by the incubation of the egg strings collected from the surviving females. Here, despite being incubated at full salinity, only 5% of the egg strings managed to hatch, and of those all produced nauplii of severely impaired viability. This result is consequent with data from Johannessen (1978), who reported that eggs of *L. salmonis* aborted and most of them died during hatching at low salinities (11.5 ‰) and with other studies stating decreased hatching of egg strings at low salinities and the sensitivity of larval stages due to their limited capacity for osmoregulation (Bricknell et al., 2006; Bron et al., 1993; Gravil, 1996a,b). Likewise, copepodid development has been reported to be inhibited at salinities <30 ‰ (Johnson & Albright, 1991; Sutherland et al., 2012), even if detrimental effects may be reversed if exposure is short term (Bricknell et al., 2006).

### 4.2 Heat challenge experiment

Our initial pilot study demonstrated that the experimental approach chosen satisfied the trade‐off between the need to accurately assess the effect of temperature on family survival, and the preservation of DNA required for parentage testing both dead and surviving lice (Supplementary file). The heat challenge was conducted in vitro*,* and therefore, we cannot predict the exact outcome from an in vivo trial. However, from a practical point of view, lice removed from their host by temperature treatments on salmon farms have negligible chances to re‐attach and will be filtered out the system. Thus, the heat challenge protocol implemented here provided a realistic challenge to simulate the outcome expected from using such treatments on a commercial farm (Grøntvedt et al., 2015; Havardsson, 2013).

Temperature strongly influences life‐history traits in ectotherms (Angilletta et al., 2004). Although the optimum temperature range for the salmon louse is not fully elucidated, it probably requires temperatures of ≥4°C to complete its life cycle successfully (Boxaspen & Naess, 2000), and it is known that at 3°C, larvae may fail to reach the infective stage (Samsing et al., 2016). Likewise, the effects of high temperature are poorly documented, but during summer 1997, the parasite was absent from Norwegian salmon farms when water temperatures exceeded 18°C (Boxaspen, 2006). It was not our goal to quantify the upper thermal limit for the salmon louse, but to investigate among‐family survival in response to thermal conditions that are known to be detrimental, and probably lethal. As for the salinity experiment, we observed significant differences in family survival. However, in contrast to the salinity experiment, background mortality was completely controlled for in this experiment. Thus, we conclude that this result demonstrates genetic variation for high‐temperature tolerance in this species. This is consistent with investigations completed in the copepod *Acartia tonsa,* which shows a significant up‐regulation of the expression of Hsp70 and Hsp90 after heat shock with particularly higher levels in individuals cultivated at 10‰ salinity sea water *versus* those at 32‰ (Petkeviciute, Kania, & Skovgaard, 2015).

### 4.3 Evolutionary implications: evolving resistance to nonchemical agents

The evolution of resistance to biocontrol agents (e.g. insecticides, fungicides and antibiotics) is a universal phenomenon that has been widely documented in the literature and constitutes a paradigmatic example of human‐induced evolutionary changes (e.g. Hemingway, Field, & Vontas, 2002; Lebarbenchon, Brown, Poulin, Gauthier‐Clerc, & Thomas, 2008; Palumbi, 2001). Likewise, salmon lice have also acquired resistance to different agents such as organophosphates (azamethiphos, dichlorvos) (Fallang et al., 2004; Jones et al., 1992), pyrethroids (cypermethrin, deltamethrin) (Fallang et al., 2005; Sevatdal & Horsberg, 2000), avermectin (emamectin benzoate) (Besnier et al., 2014; Espedal et al., 2013; Jones, Hammell, Dohoo, & Revie, 2012; Lees, Baillie, Gettinby, & Revie, 2008) and hydrogen peroxide (Helgesen, Romstad, Aaen, & Horsberg, 2015; Treasurer, Wadsworth, & Grant, 2000). The temporal frames of utilization of the aforementioned agents were variable, but none them exhibited a fully useful life beyond a decade, identical time span that has been reported for insects to evolve resistance to a new pesticide (National Research Council 2000), and for weeds, which typically evolve resistance within 10–25 years of deployment of an herbicide (see Palumbi (2001) for revision). This clearly illustrates the evolutionary capacity of the salmon louse, which displays rapid generation times, large population sizes and a high degree of connectivity among geographically distinct regions (Besnier et al., 2014; Glover et al., 2011).

The widespread loss in efficiency of chemotherapeutants to control lice infestations in commercial salmon farms catalysed the development of nonchemical delousing procedures such as warm‐water (Havardsson, 2013) and freshwater treatments (Reynolds, 2013). However, the rapidly expanding and widespread use of such alternative delousing methods arouses the concern that they, as has been the case for chemotherapeutants, may exert a selective pressure on the salmon louse, driving it to decreased sensitivity. The results of the present study, demonstrating an underlying genetic basis towards tolerance to high temperature, and suggesting the same for low salinity, certainly give cause for concern. This needs to be considered when implementing integrated management practices for control of this parasite. These concerns are warranted given that alleles conveying tolerance to the formerly described chemical treatments have proven to rapidly spread across the entire North Atlantic (Besnier et al., 2014), which agrees with the extremely weak genetic structure found in the amphi‐Atlantic distribution range of the species (Glover et al., 2011).

## DATA ARCHIVING STATEMENT

The raw data concerning both experiments in this study have been deposited as Supplementary File.
