# Supplementary material for: A pedigree‐based experiment reveals variation in salinity and thermal tolerance in the salmon louse, Lepeophtheirus salmonis
Source: Evol Appl. 2017 Aug 16;10(10):1007–19. doi: 10.1111/eva.12505 (PMC5680634; doi:10.1111/eva.12505)
Supplement: Supplementary file 1 [file EVA-10-1007-s001.doc]

**SUPPLEMENTARY INFORMATION**

**A pedigree-based experiment with the salmon louse, *Lepeophtheirus salmonis* reveals variation in salinity and thermal tolerance**

Lina Eva Robin Ljungfeldt, María Quintela, Francois Besnier, Frank Nilsen, Kevin Alan Glover

**MATERIAL AND METHODS**

**Animal welfare considerations and rearing conditions**

Although salmon lice belong to the systematic entities that are not protected by animal welfare legislation, the development of *L. salmonis* past the infective copepodid stage requires the attachment to a host fish. The Norwegian Animal Welfare Act strictly regulates the maintenance of fish used as hosts for salmon lice. All parts of this study were conducted in accordance with these regulations, under the application 2009/186329, in the wet laboratory of the Institute of Marine Research (IMR) in Bergen. Here, farmed Atlantic salmon (*Salmo salar* L.) in the size range of ca 200 to 700 g were used as hosts for cultivation of salmon lice and experiments involving lice infections. The fish used for culturing the lice strains were kept in 250 L and 500 L multiple-fish tanks. These were hand fed once daily on a commercial diet. Tank water exchange, aeration and current was regulated by inlet water flow kept at ca 60 L∙hr−1 per kg fish (minimum 400 L∙hr1, in order to maintain sufficient flow in the tank), of natural seawater pumped up from 120 m depth and passed through a column aerator. The water temperature was kept at 9.0 ± 1°C, and salinity at 34 ± 0.5‰ throughout the year. The tanks were kept indoors at an artificial, 12 hrs daily (fluorescent) light regime under transparent lids. The fish used as hosts during the production of full sibling salmon lice families, by pairing a single male and a virgin female louse together on a single fish, were placed individually in an array of 36 plastic tanks, each of 50 L volume. This setup was similar to that described by Hamre and Nilsen (2011), and are hereafter referred to as the single-fish tanks. All procedures involved in propagating, handling and quantification of *L. salmonis* were performed using the methods and culturing systems described in detail by Hamre et al. (2009).

**Pilot study to identify a heat-challenge challenge protocol**

*Background and method of the pilot study*

The aim of the *in vitro* pilot study was to establish a challenge-protocol that enabled exposing the lice *in vitro* to a heat-challenge that would cause selective mortality in a predictable and accurate manner, but simultaneously enabled dead lice to be rapidly sampled into EtOH to conserve DNA quality for parentage testing.

Four 1L-beakers, each containing 150 lice (two beakers representing with lice from the LsNo strain, and two beakers with lice from the LsSo strain) were incubated in a water-bath for 2 hours at 26 °C. In addition, five 1L-beakers filled with water were also incubated in the same bath to ensure having water at the same temperature. After two hours of incubation, water in the four beakers containing lice was gently vortexed and poured out into another container. Lice that were not attached to the glass sides of the beaker were thus poured out at the same time. Immediately afterwards, the empty beakers containing lice still attached to the glass walls were re-filled with water at 26 °C. Lice were classified as “attached” (the ones that remained in the walls of the beakers = presumptive survivors) or “detached” (the ones that flushed with the water to another container = presumptive mortalities). All eight beakers (i.e., those containing “presumptive survivors” and “presumptive mortalities”) were thereafter incubated at 9 °C for a further 24 hours. After 24 hours, the same process was repeated to assess how many lice remained attached to the walls of the beakers and how many of them were flushed with the poured water.

Lice were evaluated immediately after the initial temperature exposure (26 °C, 2h) as “attached” (A, the ones alive on the beaker walls) and “detached” (D, the ones that left the beaker when pouring the water out). After 24 hours of incubation at 9 °C, they were again classified in an analogous way. Thus, four categories resulted: AA (individuals correctly identified as survivors from the beginning), AD (attached first and then detached; *i.e.* false positives), DA (detached first and re-attached; *i.e*. false negatives) and DD (correctly identified as dead in the first instance).

*Results of the pilot study*

The vast majority of the lice identified as presumptive survivors after the initial 2-hour incubation at 26 °C (i.e. still attached to the beaker walls) were still attached to the walls of the beakers 24 hours later (Table S2). Thus, there is good reason to suggest that these individuals were indeed true survivors of the heat-challenge treatment. Approximately, half of the lice classified as presumptive mortalities after the initial 2-hour incubation at 26 °C (i.e. detached from the beaker walls) were found re-attached to the beaker wall 24 hours later, while the other half of these presumptive mortalities had reattached to the beaker wall after 24 hours (Table S2). Thus it can be approximately stated that determination of presumptive mortalities, in contrast to determination of survivors, was only 50% correct after the initial 2-hour heat-challenge challenge. However, and this is a vitally important detail, in the wild, pre- or adult lice that detach from a salmonid host will most likely not have the opportunity to find another host (either in the wild or in a farm). Thus, while it can be argued that approximately 50% of the presumptive mortalities from the 2-hour heat-challenge may have recovered from the treatment, if given an available host to re-attach to, in the wild, or more relevant, in a farming situation where heat is used as a delousing treatment, these individuals will be collected from the filter water and will not have had the chance to re-find a host. Thus, a 2-hour exposure at 26 °C using the protocol described here provides a reliable and accurate way to differentiate lice in their ability to tolerate a temperature shock.

**REFERENCES**

Hamre, L. A., K. A. Glover, and F. Nilsen. 2009. Establishment and characterisation of salmon louse (*Lepeophtheirus salmonis* (Krøyer 1837)) laboratory strains. *Parasitology International* 58 (4):451-460.

Hamre, L. A. & F. Nilsen. 2011. Individual fish tank arrays in studies of *Lepeophtheirus salmonis* and lice loss variability. *Diseases of Aquatic Organisms* 97 (1):47-56.

**TABLES AND FIGURES**

**Table S1**. Incubation of egg strings recollected from 100 females surviving after the heat-challenge experiment. DPH stands for “Days Post Hatching” and describes the age of copepodids at infection time. Number of incubated egg string pairs, number of nauplii that hatched from each family, and number (and percentage) of alive nauplii.

| **Family** | **DPH** | **N egg string pairs** | **N nauplii** | **Alive nauplii** |
| --- | --- | --- | --- | --- |
| Fam-LsB09 | 7 | 13 | 343 | 0 (0) |
| Fam-LsB10 | 7 | 17 | 205 | 9 (4.39) |
| Fam-LsB12 | 9 | 7 | 89 | 4 (4.49) |
| Fam-LsS09 | 7 | 7 | 43 | 0 (0) |
| Fam-LsS10 | 7 | 7 | 98 | 7 (7.14) |
| Fam-LsS11 | 7 | 11 | 388 | 31 (7.99) |
| Fam-LsS12 | 6 | 4 | 0 | 0 (0) |
| Fam-LsS13 | 7 | 11 | 118 | 3 (2.54) |
| Fam-LsS14 | 8 | 23 | 404 | 0 (0) |

**Table S2**. Summary tables of GLMM outputs reporting the variance contribution of each random factor.

| **Experiment** | **Random factors** | **Variance estimate ± SD** | **P-value** |
| --- | --- | --- | --- |
| Salinity | Type | 0 ± 0 | 0.99 |
|  | Family | 0.70 ± 0.84 | <0.001 |
|  | Tank | 0 ± 0 | 0.59 |
|  | Sex | 0.31 ± 0.17 | 0.001 |
| Heat-challenge | Type | 9.8 10^-9^ ± 9.9 10^-5^ | 0.03 |
|  | Family | 0.18 ± 0.43 | <0.001 |
|  | Tank | 0.45 ± 0.67 | <0.001 |
|  | Sex | 0.16 ± 0.40 | <0.001 |

**Table S3**. Pilot study prior to heat-challenge experiment: Four experimental containers of lice from LsNo and LsSo strains followed this experimental sequence: a) exposure at 26 °C for 2 hours, b) evaluation to assess attached/detached individuals, c) incubation at 9 °C for 24 hours, and d) re-evaluation. The four resulting categories correspond to: AA (individuals correctly identified as survivors from the beginning), AD (attached first and then detached; *i.e.* false positives), DA (detached first and re-attached; *i.e.* false negatives) and DD (correctly identified as dead from the beginning). Numbers (and percentage) of individuals are indicated in each case.

|  | **Lice strain** | | | |
| --- | --- | --- | --- | --- |
| **Categories** | **LsNo** | **LsNo** | **LsSo** | **LsSo** |
| Attached – Attached (AA) | 72 (49.32) | 78 (49.06) | 51 (31.88) | 65 (45.14) |
| Attached – Detached (AD) | 0 (0.00) | 4 (2.52) | 2 (1.25) | 8 (5.56) |
| Detached – Attached (DA) | 31 (21.23) | 40 (25.16) | 49 (30.63) | 41 (28.47) |
| Detached – Detached (DD) | 43 (29.45) | 37 (23.27) | 58 (36.25) | 30 (20.83) |
| Total | **146** | **159** | **160** | **144** |

**FIGURE LEGENDS**

**Fig. S1.** Map of the sampling locations for the lice strains used in the salinity (LsS and LsB) and the heat-challenge (LsSo and LsNo) experiments.

**Fig. S2**. Annual sea water temperature variations over ten years prior to sampling, from two hydrological stations close to the areas were the two louse strains used in the heat-challenge trial were sampled: A) Ingøy hydrological station, Finnmark (LsNo), and B). Sognesjøen, Sogn og Fjordane (LsSo).

**Fig. S3.** *L.salmonis* individuals at different life cycle stages presented for comparison of body sizes. Top row: females. From left to right – one newly moulted adult female (before the elongation of the genital segment), two groups of seven preadult II and nine preadult I individuals, respectively. Bottom row: males. From the left– two groups of six adult and twelve preadult II individuals, and one preadult I male to the right. Note that, disregarding not being at the same life-stage, the adult males and the preadult II females are of approximately the same body size. Due to staggered developmental rates between the genders (at least in later life stages), male lice reach the adult stage faster than the females, thereby, through synchronized infections under the same environmental conditions there is a time frame (after the last moult of the males and before the last moult of the females) where it is possible to compare lice of different gender that are demonstrably of the same age (actual *and* physiological) and size (approximately). Using this opportunity will reduce the risk of interpreting gender related differences from responses that are actually biased or structured by age or size of the test individuals.
