# Supplementary figures and images for: A pedigree‐based experiment reveals variation in salinity and thermal tolerance in the salmon louse, Lepeophtheirus salmonis
Source: Evol Appl. 2017 Aug 16;10(10):1007–19. doi: 10.1111/eva.12505 (PMC5680634; doi:10.1111/eva.12505)

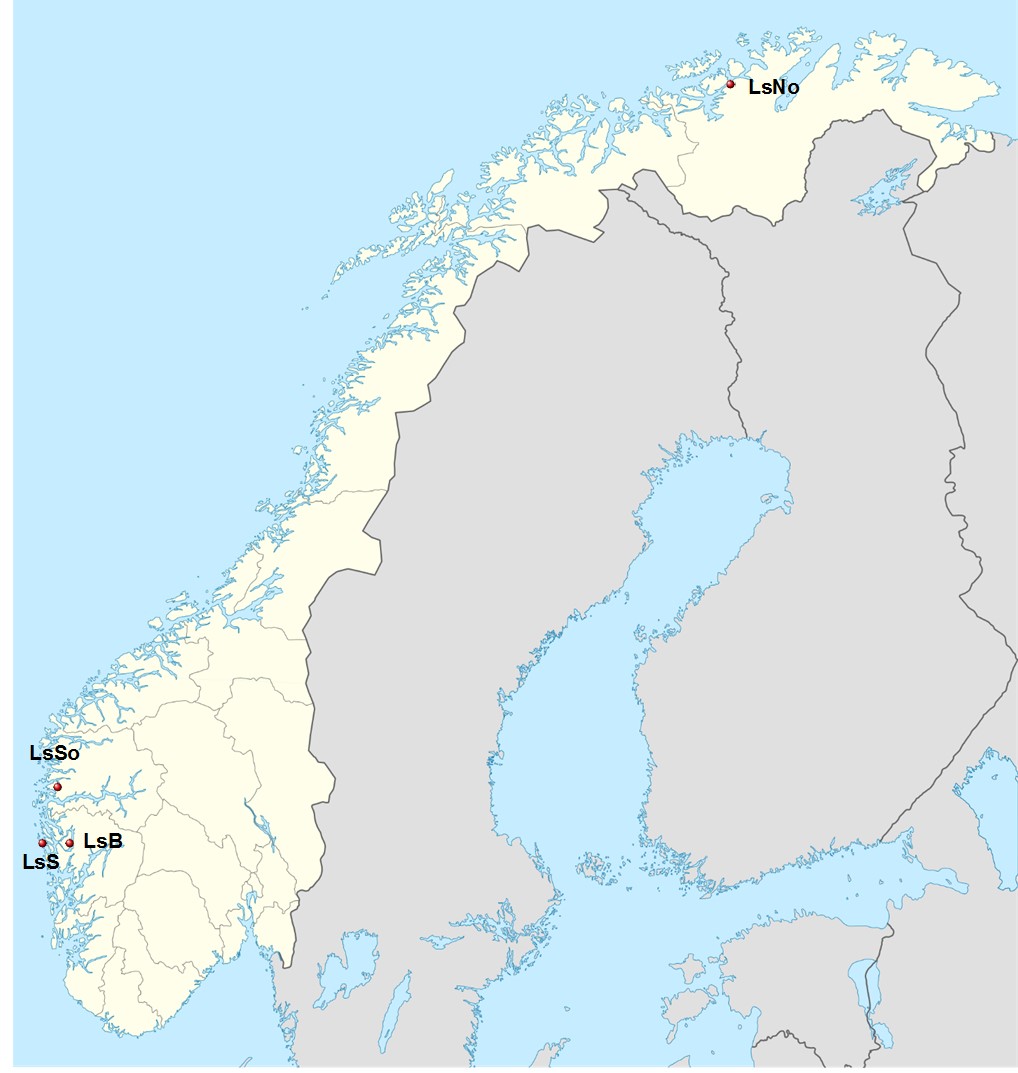

Supplement: Supplementary file 2 [file EVA-10-1007-s002.jpg]

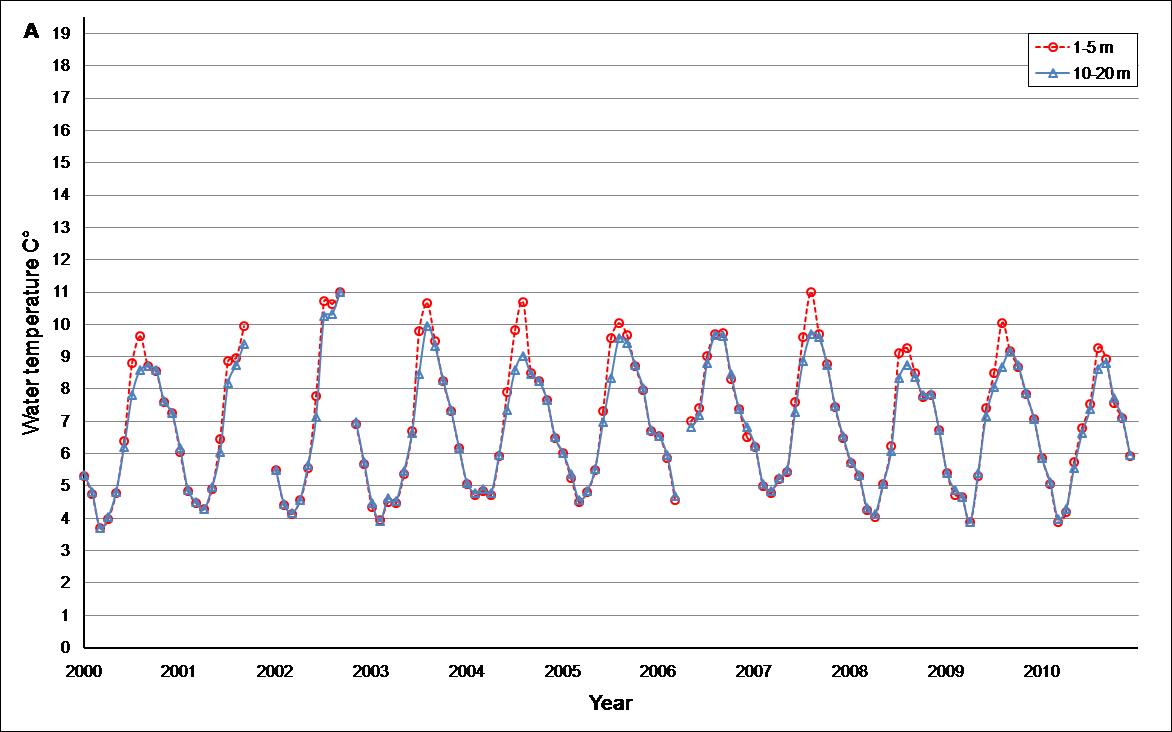

Supplement: Supplementary file 3 [file EVA-10-1007-s003.jpg]

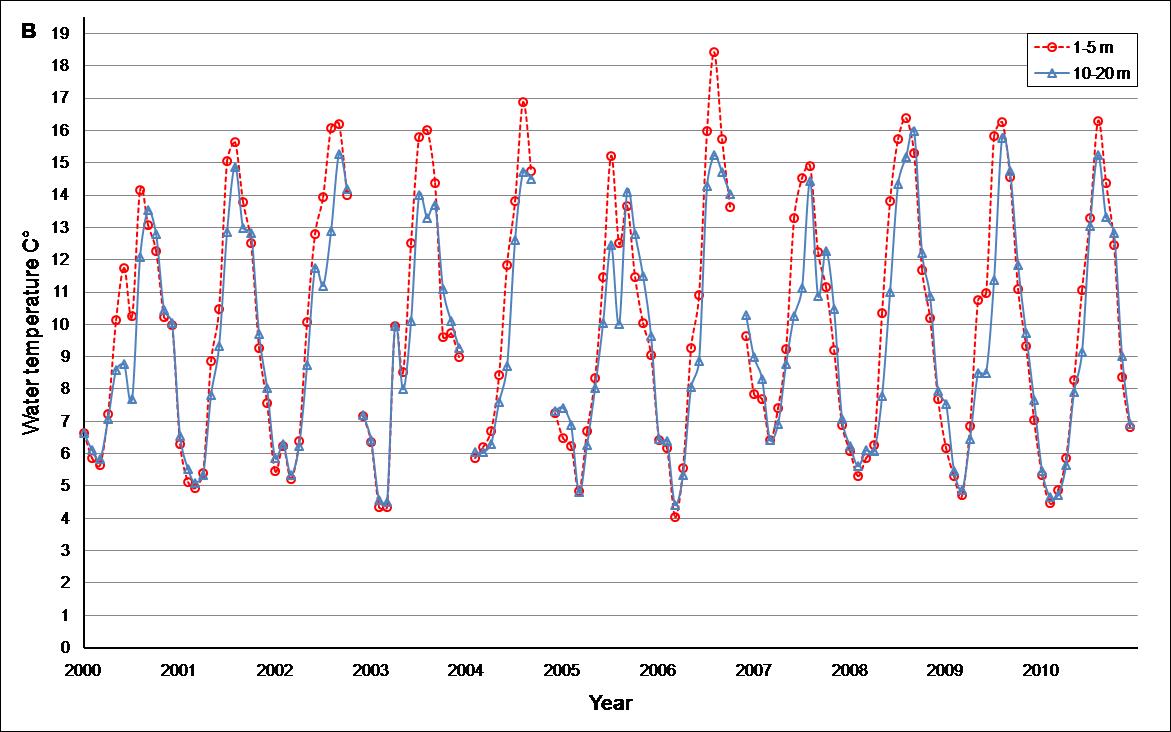

Supplement: Supplementary file 4 [file EVA-10-1007-s004.jpg]

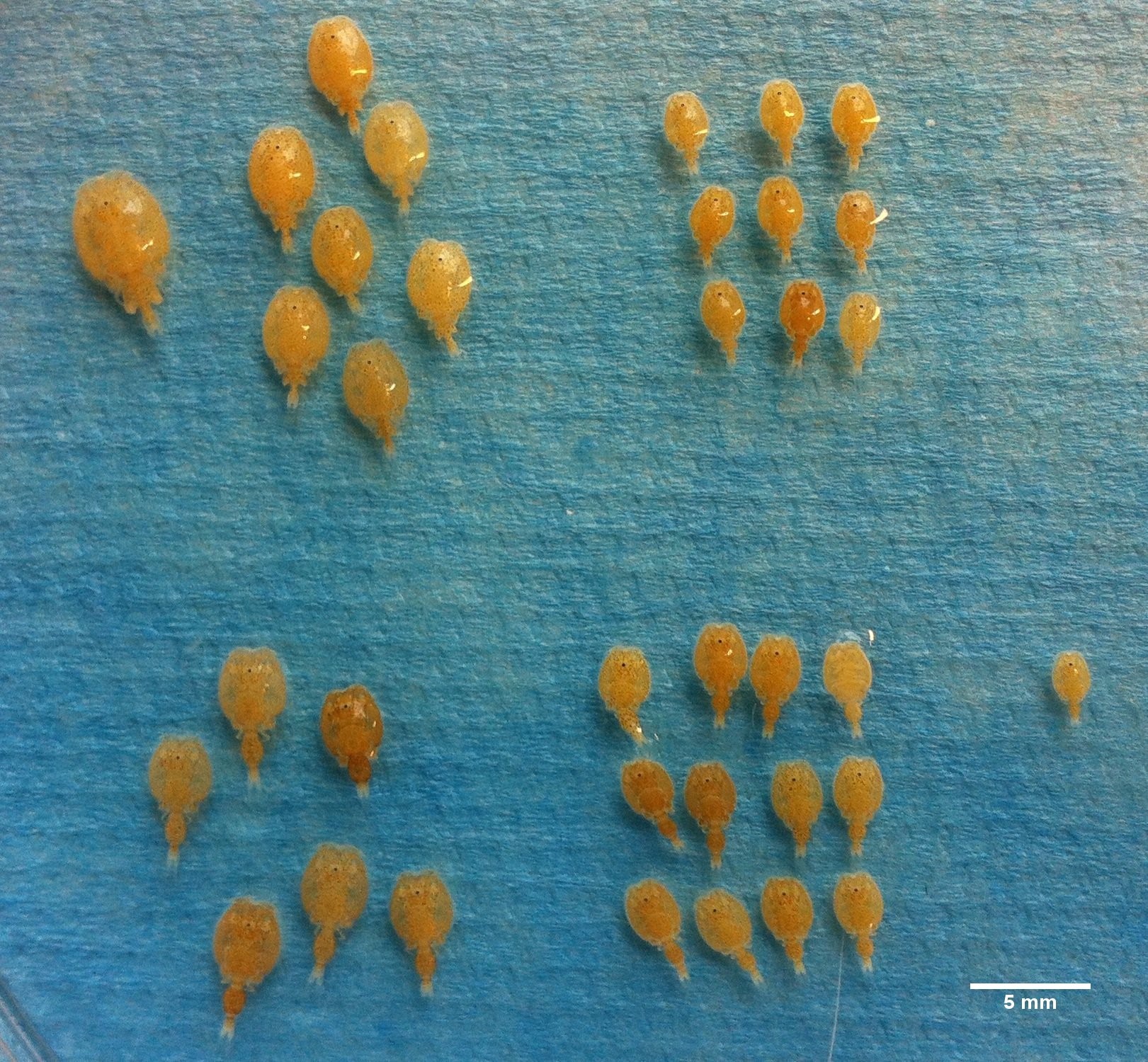

Supplement: Supplementary file 5 [file EVA-10-1007-s005.jpg]
